# Endo180 at the cutting edge of bone cancer treatment and beyond†

**DOI:** 10.1002/path.4673

**Published:** 2016-01-11

**Authors:** Justin Sturge

**Affiliations:** ^1^ School of Biological, Biomedical and Environmental SciencesUniversity of HullUK

**Keywords:** bone, collagen, Endo180, metastasis, metastatic, osteoblast, osteoid, osteolytic, osteolysis, osteosarcoma, therapy

## Abstract

Skeletal bone is an attractive site for secondary tumour growth and is also home to spontaneous primary cancer. Treatment of bone metastasis is focused on limiting the vicious cycle of bone destruction with bisphosphonates or inhibition of receptor activator of nuclear factor‐κB ligand (RANKL) with the fully human monoclonal antibody denosumab. The estimated 1 million deaths/year where bone metastasis is present, and the high healthcare costs required for its management, have ignited intensive research into the cellular and molecular pathology of osteolysis, involving interplay between tumour cells, bone‐forming osteoblasts and bone‐degrading osteoclasts. Compared to bone metastasis, knowledge about the pathology of primary bone cancers is limited. In recent work published in this journal, Engelholm et al provide a unique insight into how this poorly understood disease manifests and destroys bone. For the first time they have demonstrated that a mouse monoclonal antibody targeting the collagen receptor Endo180 (CD280, MRC2 uPARAP) can prevent osteolysis and bone destruction in a syngeneic model of advanced osteosarcoma. Their convincing findings make an important contribution towards Endo180‐based therapy being developed as an option for the treatment of bone cancer amongst other malignancies. © 2015 The Authors. *The Journal of Pathology* published by John Wiley & Sons Ltd on behalf of Pathological Society of Great Britain and Ireland.

Engelholm *et al* have taken significant steps towards Endo180 taking its place centre stage as a *bona fide* cancer target 1. Endocytic receptor 180 was identified by Isacke *et al* in 1990 as an endocytic receptor expressed by stromal cells 2. In 2000 the full‐length human Endo180 cDNA clone was isolated 3 and the receptor was validated to be a novel urokinase plasminogen activator‐associated protein (hence its alternative name, uPARAP) 4. The biological roles of Endo180 include extracellular matrix (ECM) remodelling as a result of its interaction with collagen via its fibronectin type II domain (FNII) 5 and its capacity to promote cell‐migratory signalling pathways and invasiveness 6, 7, 8, 9. In osteosarcoma cells, Endo180 is strongly localized to sites of cell–matrix contact and plays a fundamental role in promoting their migration via the Rho–ROCK pathway 7. At the tissue level in bone, Endo180 is localized to areas of active remodelling 10, 11 and genetic silencing or mutation of Mrc2 results in some dramatic skeletal defects 11, 12, 13.

Engelholm *et al*'s study shines a spotlight upon the pathology of osteosarcoma 1. Osteosarcoma is the most frequent primary bone cancer (incidence 0.2–3/100 000/year) that accounts for >10% of all solid cancers in adolescents (incidence 0.8–11/100 000/year at age 15–19). It normally arises in the metaphysis of long extremity bones, typically in the vicinity of the knee, and is four times more likely to affect males as females. The primary hallmark of osteosarcoma is the production of excessive bony osteoid matrix by the malignant cell population. Osteosarcoma can be osteoblastic, chondroblastic and fibroblastic in origin, or can be a secondary complication of Paget's disease, or the result of radiation therapy used for a pre‐existing pathology. Localized osteosarcoma can be treated by chemotherapy combined with function‐preserving surgery. If osteosarcoma metastasizes to other parts of the bone, it becomes inoperable and the only therapeutic option at this advanced stage of the disease is chemotherapy. Targeted treatments that can limit osteosarcoma progression do not exist.

In their paper, Engelholm *et al* explore the role of Endo180 as a putative therapeutic target in osteosarcoma 1. They meticulously analysed human osteosarcoma tissue samples by specifically focusing their attention on the malignant cells located at the ‘cutting edge’ of the tumour mass where osteolytic activity helps to create new space for growth and expansion. By zooming into these areas, they made an intriguing observation regarding the potential cellular mechanisms involved in disease progression. In contrast to the high numbers of CD68‐positive osteoclasts with high tartrate‐resistant acid phosphatase (TRAP) activity seen at the cutting edge of secondary bone lesions derived from solid tumours, these highly specialized bone‐degrading cells could not be found. Instead the osteolytic areas of the tumours were abundant with Endo180‐positive and MT1‐MMP‐positive osteosarcoma cells that formed layers in direct contact with the bone surface.

Injection of the osteosarcoma cell line (NCTC‐2472) into the femurs of mice was used to achieve high levels of osteolytic activity. This syngeneic mouse model recapitulated the observations made in human specimens, with abundant layers of Endo180/MT1‐MMP‐positive NCTC‐2472 cells sitting on bone surfaces that were virtually devoid of osteoclasts. Treatment with the monoclonal antibody (mAb) 5f4, which recognizes an epitope in the first three N‐terminal domains of Endo180 [cysteine‐rich domain (CRD), FNII and the first C‐type lectin domain (CTLD) out of eight, CTLD1 (Figure 1)], silences Endo180 by an unknown mechanism 14 and blocked the uptake of fluorescently labelled protein fragments released from bovine bone slices by NCTC‐2472 cells. The bony fragments were traced to lysosomes, to which collagen internalized by Endo180 is trafficked via an endocytic pathway for its degradation. Moreover, in the syngeneic mouse model, the Endo180‐silencing mAb 5f4 protected femoral bone against the osteolytic destruction induced by the presence of NCTC‐2472 cells. These convincing data add significant weight to a growing body of evidence that dysregulated Endo180‐dependent mechanisms, in tumour cells and tumour‐associated stromal cells, play central roles in bone cancer and other malignancies 15.

**Figure 1:**
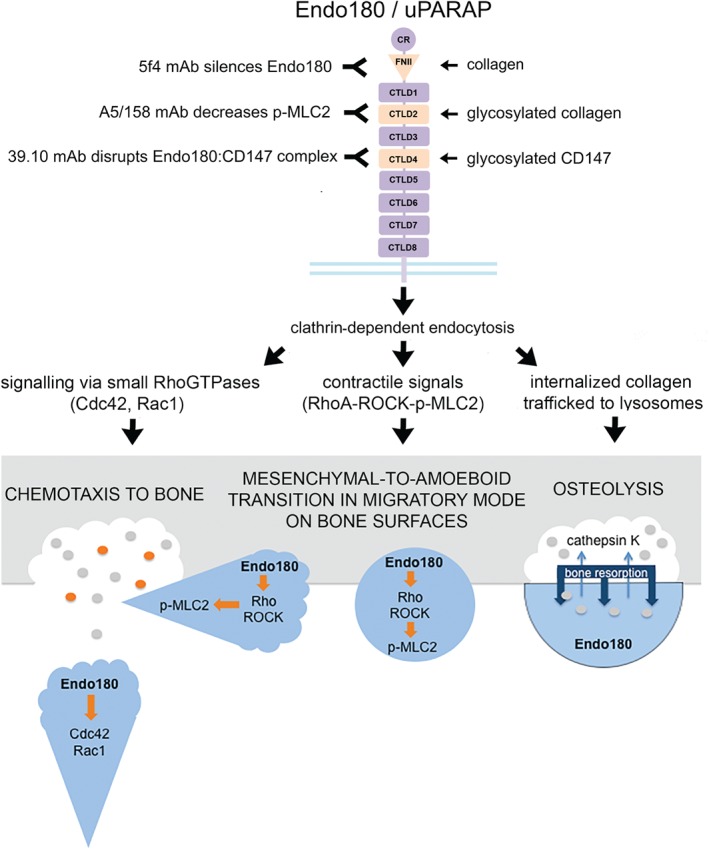
Antibody targeting of Endo180/uPARAP in cancer. The Endo180 ectodomain is composed of cysteine‐rich (CR) and fibronectin type II (FNII) domains followed by eight C‐type lectin domains (CTLD1‐8) 3. The FNII domain binds to collagen 5, CTLD2 binds to glycosylated collagen 9 and CTLD4 binds to glycosylated CD147 8. Endo180 is internalized from the plasma membrane via clathrin‐dependent endocytosis 2, 3. Endosomal Endo180 is responsible for: (a) spatiotemporal activation of contractile signals via the phosphorylation of myosin light chain‐2 (p‐MLC2) 7, which drives cell migration and invasion; (b) signalling via the small RhoGTPases Cdc42, Rac1 and RhoA 6 and chemotaxis towards gradients of urokinase plasminogen activator (uPA) 6 or collagen fragments 20; and (c) trafficking of internalized collagen to lysosomes for degradation during osteolysis 1. The effects of monoclonal antibody (mAb) blockade of Endo180 are shown, including: (a) 5 F4 mAb, which binds to an epitope in the first three domains of the receptor (CR, FNII, CTLD1) and silences Endo180 via an unknown mechanism 14; (b) A5/158 mAb, which binds to an epitope in CTLD2 6 and decreases phosphorylated myosin light chain (p‐MLC2) 7, 9; and (c) 39.10 mAb, which binds to an epitope in CTLD4 and disrupts its binding to CD147 8. 5f4 mAb has been shown to block osteolysis induced by osteosarcoma cells 1; A5/158 mAb blocks chemotaxis towards uPA in adenocarcinoma cells 6 and decreases p‐MLC2 induced by a stiff microenvironment in 3D epithelial cell acini 9; and 39.10 mAb blocks chemotaxis towards uPA in adenocarcinoma cells 6 but induces invasive behaviour in 3D epithelial cell acini 8. The different roles of Endo180 in osteolytic bone metastasis that could be targeted using antibody‐based therapy, depicted in the schematic, include chemotaxis of tumour cells to bone 6, 20, mesenchymal‐to‐amoeboid transition in tumour cell migration on bone surfaces 21 and tumour cell‐associated osteolysis 1

In accordance with the findings of Engelholm *et al* 1, targeting Endo180 by genetic silencing or treatment with the neutralizing mAbs A5/158 and 39.10 can block the migratory behaviour of osteosarcoma and other invasive tumour cell types 6, 7 (Figure 1). On the flipside, epitope‐specific targeting of the cell–cell adhesion function of the fourth CTLD (CTLD4) in Endo180, using the mAb 39.10 or a dominant negative CTLD4 protein construct, was found to be sufficient to induce invasive behaviour in normal glandular epithelial cells via disruption of the epithelial‐to‐mesenchymal (EMT) suppressor complex that it forms with the extracellular matrix metalloproteinase inducer (EMMPRIN; CD147) 8. Similar to the blockade of osteosarcoma progression in femoral bone by the mAb 5f4, the *de novo* invasion of prostate epithelial cells induced by exposure to a stiff microenvironment can be blocked by the mAb A5/158, which recognizes an epitope in the second CTLD (CTLD2) of Endo180 9. The clinical impact that this therapeutic strategy could have is clear from the significant survival benefit of Endo180‐negative versus Endo180‐positive tumours in prostate cancer patients 8, 9. Together with the work of Engelholm *et al* 1, these findings suggest that targeting epitopes in the N‐terminus of Endo180 (CRD, FNII, CTLD1, CTLD2), using an antibody‐based therapeutic approach, should be considered for the treatment of malignancies associated with the promotion of mechanotransduction pathways, including the phosphorylation of myosin light chain‐2 (p‐MLC2), in stiff tissues such as the ageing prostate gland and bone (Figure 1).

The mechanism of tumour‐mediated bone degradation proposed by Engelholm *et al* pinpoints the malignant cells in the tumour mass as the major participants in osteolysis 1. This finding contrasts with the pathology that underlies the establishment and progression of bone metastasis, which involves vicious cycles of bone destruction driven by paracrine and autocrine signals in adjacent tumour cells, osteoblasts and osteoclasts 16. Tumour cells that establish niches in the bone interact with adjacent bone‐forming osteoblasts and bone‐degrading osteoclasts, disrupting normal bone homeostasis, where levels of its formation and degradation are maintained in equal balance. By using an *in vitro* model to recapitulate the bone‐forming side of this complex setting, Endo180 was identified as a modulator of mineralized type I collagen deposition in primary human osteoblasts that is activated by the transforming growth factor‐β1 (TGFβ1) and TGFβ1 receptor (TGFβ1R) signalling axis and suppressed by paracrine signals from adjacent tumour cells 17. Given that the primary hallmark of human osteosarcoma is excessive mineralized type I collagen deposition, a reduction in its production by the targeted blockade of Endo180 function may also contribute to limiting the bony deposits made by osteosarcoma cells and osteoblasts associated within the tumour mass and adjacent bone stroma 17. A clearer understanding of how the Endo180 pathway regulates osteoid deposition in primary and secondary bone cancer will require its in‐depth evaluation as an anti‐osteogenic target in models that recapitulate their dominant osteosclerotic nature.

Osteolysis mediated by osteoclasts requires the formation of sealing zones on the bone surface and the localized release of cathepsin K, which is the only enzyme capable of degrading the mineralized type I collagen matrix of bone. In support of the model proposed by Engelholm *et al*, cathepsin K expression has been demonstrated in osteoblasts and tumour cells associated with osteosarcoma, giant cell tumour of bone and prostate cancer 18. From a pathological perspective, it will be interesting to explore how Endo180 cooperates with cathepsin K in the contexts of primary and secondary bone cancers; however, it would be unlikely that a combined therapeutic approach incorporating cathepsin K inhibitors would be considered, given their withdrawal from further clinical evaluation due to their severe side‐effects 16. A better therapeutic option would be neo‐adjuvant therapy combining an Endo180‐targeted antibody and bisphosphonates. Interestingly, bisphosphonates significantly decrease Endo180 levels in the serum of patients with advanced breast cancer and bone metastasis 19, suggesting that they are associated with a common pathological pathway and may provide an additive therapeutic advantage if co‐administered. As for the therapeutic response to bisphosphonates, responses to Endo180‐targeted therapy could be monitored using serum levels of soluble Endo180.

Endo180‐dependent chemotaxis towards collagen fragments or urokinase plasminogen activator (uPA) is linked respectively to osteoblast recruitment to sites of bone degradation under normal physiological conditions 20 and the migratory behaviour of metastatic tumour cells 6. Once in contact with human bone surfaces, tumour cells undergo a mesenchymal‐to‐amoeboid transition in their mode of migration that is driven by Endo180 21. The same scenario could help the spread of osteosarcoma, other primary bone cancers and secondary bone metastases originating from solid tumours, whereby the excessive release of collagen fragments and other peptides at the cutting edge of the tumour mass, or premetastatic niches, might act as chemotactic signalling cues to coordinate their invasive spread towards these secondary sites and their transition to the highly invasive amoeboid (rounded) mode of migration (Figure 1). Blocking this process with Endo180‐targeted antibodies could be another key to preventing the homing of primary bone cancer cells, and cells disseminating from primary tumours, towards secondary sites in the bone.

In a snapshot, the advances that have been made since Endo180 was first isolated 25 years ago have led us towards the exciting findings of Engelholm *et al* 1 reported in this issue. Over the next few years we will hopefully be able to see Endo180‐based therapeutics and diagnostics translate into improved cancer treatment.
